# Beyond a trauma-informed approach and towards shame-sensitive practice

**DOI:** 10.1057/s41599-022-01227-z

**Published:** 2022-06-24

**Authors:** Luna Dolezal, Matthew Gibson

**Affiliations:** 1 University of Exeter, Exeter, UK; 2 University of Birmingham, Birmingham, UK

## Abstract

In this article, we outline and define for the first time the concept of shame-sensitivity and principles for shame-sensitive practice. We argue that shame-sensitive practice is essential for the trauma-informed approach. Experiences of trauma are widespread, and there exists a wealth of evidence directly correlating trauma to a range of poor social and health outcomes which incur substantial costs to individuals and to society. As such, trauma has been positioned as a significant public health issue which many argue necessitates a trauma-informed approach to health, care and social services along with public health. Shame is key emotional after effect of experiences of trauma, and an emerging literature argues that we may ‘have failed to see the obvious’ by neglecting to acknowledge the influence of shame on posttrauma states. We argue that the trauma-informed approach fails to adequately theorise and address shame, and that many of the aims of the trauma-informed are more effectively addressed through the concept and practice of shame-sensitivity. We begin by giving an overview of the trauma-informed paradigm, then consider shame as part of trauma, looking particularly at how shame manifests in post-trauma states in a chronic form. We explore how shame becomes a barrier to successful engagement with services, and finally conclude with a definition of the shame-sensitive concept and the principles for its practice.

## Introduction

*“Shame has ruled my whole life”* – Anonymous, trauma survivor

*“Trauma leads to shame. Trauma determines the content of shame. Shame pushes the body into a traumatic response. The more I learn about the two, the more I am convinced of their deep connection to one another.”* – Lucia Osborne-Crowley (Osborne-Crowley, 2020)

Experiences of trauma are widespread, and there exists a wealth of evidence directly correlating trauma to a range of poor social and health outcomes which incur substantial costs to individuals and to society. As such, trauma has been positioned as a significant public health issue which, as Magruder et al. (2017) argue, necessitates a ‘trauma-informed approach’ (TIA) to public health policy agendas. Shame is key emotional aftereffect of trauma, and an emerging literature argues that we may “have failed to see the obvious”by neglecting to acknowledge the influence of shame on post-trauma disorders (Taylor, 2015). In this article, we argue that effectively addressing the post-traumatic state necessitates a clear understanding of shame, its phenomenology and its effects. We demonstrate that shame is a core aftereffect of traumatic experiences and argue that being sensitive to shame addresses many issues related to trauma, while also supporting good practice for all that come into contact with human services. We outline and define for the first time the concept of shame-sensitivity and the principles for shame-sensitive practice. We begin by giving an overview of the trauma-informed paradigm, then consider shame as part of trauma, looking particularly at how shame manifests in the post-traumatic state in a chronic form. We explore how shame becomes a barrier to successful engagement with services, and finally conclude with a definition of the shame-sensitive concept and the principles for its practice. Offering strategies for shamesensitive practice, this article highlights the need for shame competence in health, care and social services.

### The trauma-informed approach

While trauma has been studied for over one hundred years it was not until the 1980s and 1990s that the topic had sufficient inter-disciplinary support to develop into a field of research and produce a theory of trauma. While there is no unified approach or understanding of trauma, most agree that it entails an event that involves “threats to life or bodily integrity, or a close personal encounter with violence and death” (Herman, 1992, p. 33), and that the experience of this event is overwhelming, resulting in long lasting effects which can encompass significant alterations to one’s experience of self, others and the world (SAMHSA, 2014). Particularly significant are experiences of trauma in early life, or Adverse Childhood Experiences (ACEs), such as abuse, deprivation, violence, witnessing of violence, neglect and disrupted attachment, among others (Poole and Greaves, 2012). Also significant are experiences of trauma in later life, such as interpersonal violence, sexual assault, warfare, tyranny under oppressive regimes, natural disasters, domestic abuse, among many others (Pattison, 2000, p. 96). While trauma can lead to post-traumatic stress disorder (PTSD) or other trauma or stressor-related disorders, which are classified as psychopathologies in the *Diagnostic and Statistical Manual 5^th^ Edition* (DSM-V), not all post-trauma states or experiences warrant being classified as pathological or fall under the umbrella of a disorder. Nonetheless, research demonstrates that individuals who have experienced trauma can have adverse outcomes in all areas of life, and that these effects can endure across a lifetime.

The interest in trauma, and its links to health and social outcomes, increased following the publication of the Felitti et al. (1998) paper on ACEs. With a sample of close to ten thousand, it is one of the largest investigations of childhood abuse and neglect, concluding that there is a strong relationship between “the breadth of exposure to abuse or household dysfunction during childhood and multiple risk factors for several of the leading causes of death in adults” (Felitti et al., 1998, p. 245). This study has been influential in subsequent research into trauma and the development of policy for services that seek to address issues related to adversity and trauma. There is now a large body of research that demonstrates that individuals who have experienced trauma can have adverse outcomes in all areas of life, and that these effects can endure across a lifetime. These individuals are significantly more likely to suffer from a range of “social, psychiatric, psychological, behavioural and physical problems” (Knight, 2019, p. 80), such as chronic health issues, mental health problems and substance use problems, as well as being correlated with social outcomes such as homelessness, violence, marital problems and incarceration, among others (Banaj and Pellicano, 2020).

The term “trauma-informed” was introduced by Harris and Fallot in 2001 as a means to integrate an understanding of trauma and its aftereffects into mental health services, following the evidence that a significant number of individuals accessing mental health services were survivors of physical and sexual abuse (Harris and Fallot, 2001). Adopting a TIA attempts to embed an understanding of how experiences of trauma can become central to an individual’s life course and life outcomes, having a profound negative effect on social outcomes, emotional wellbeing, mental and physical health, along with health-relevant behaviour (Poole and Greaves, 2012), impeding an individual’s ability to seek out and engage with health and social services that are designed to help them (Barrett, 2019). TIAs involve a paradigm shift in how services and professionals respond to patients and clients, attempting to address root causes rather than surface symptoms, reframing the core diagnostic question from enquiring, “What is wrong with you?” to understanding, “What happened to you?” (Kimbery and Wheeler, 2019, p. 42; SAMHSA, 2014). This approach recognises that “any person seeking services or support might be a trauma survivor” and that “systems of care need to recognise, understand and counter the sequelae of trauma to facilitate recovery” (Goodman et al., 2016, p. 748).

Central to the TIA is an understanding that typical emotional, psychological and social aftereffects of trauma directly impede an individual’s ability to seek out and engage with the human services that are designed to help them (Barrett, 2019). In addition, when trauma survivors do manage to engage with the services that may help them, the interactions they have with organisations, staff and care providers, who do not recognise and understand their trauma and its aftereffects, may inadvertently lead to a further disengagement and entrenchment of the problems (e.g., substance use, mental ill health) that these services are designed to diagnose and treat. The central contention of the TIA is that applying a ‘trauma lens’ can powerfully elucidate the root causes of ill health, health-related behaviours and social difficulties, leading to more effective interventions, support, diagnoses and treatments. This has led to the redesigning and reconceptualization of some health, care and social services, using the TIA paradigm as a way to structure the way that care is delivered (Gerber, 2019; SAMHSA, 2014; Wilson et al., 2013).

In a Western context, TIA has gained influence in international policy making circles. For example, in the United States there are many programmes designed to integrate the TIA at federal, state and community levels (Melz et al., 2019). Within the United Kingdom, the Scottish and Welsh Government are seeking to develop and integrate the TIA into a range of public services. (Scottish Government, 2020; Welsh Government, 2021). This is equally the case in England, with Plymouth leading the way by seeking to become the United Kingdom’s first ‘trauma informed city’ (Plymouth City Council, n.d.). The TIA is not only being advanced geographically but also practically, being applied to an ever-greater range of public services including children and youth, education, and health services, probation, and policing.

### Critiques and limitations

The TIA is not without criticism. Conceptually, ‘trauma’ is a farranging concept that covers a wide range of experiences, and also a broad spectrum of outcomes. In considering how the concept of ‘trauma’ has been advanced in the TIA, Wastell and White (2017) argue that there are fundamental problems with how original research on trauma experiences has been interpreted for policy and practice. They argue that the original science underpinning our understanding of trauma expresses uncertainty and tentative conclusions, but that this inconclusiveness has been removed in the translation to practice in the TIA, resulting in definitive answers and concepts that are no longer consistent with the foundations of trauma research. Their concerns raise important conceptual and philosophical questions regarding how trauma is defined and understood, and how this is translated into practice.

Equally, there are conceptual implications as a result of the link between trauma and the original ACEs study. As the concept of trauma was boosted by the publication and promotion of the ACEs study, the case for the TIA is often justified by the research on ACEs. However, as Berliner and Kolko (2016) argue, not all harmful or stressful life experiences that the ACEs study examined were traumas; the two are not synonymous. Furthermore, there are those who have criticised the concept of adversity used in the original ACEs study to argue that not only do the components fail to identify adverse experiences (a parental separation is considered an adverse experience when this could be a protective one, for example) but that it is also a very narrow concept that misses many other forms of adversity, particularly wider individual, social and community forms of adversity such as chronic illness, or on-going social harms like poverty, deprivation or discrimination (White et al., 2019). There are on-going academic and practical debates relating to how to address the effects of trauma and ACEs. For example, Steptoe et al. (2019) argue there is a need for more information on approaches that address ACEs, while Asmussen et al. (2019) review a range of interventions that seek to address ACE-related trauma. To address such criticisms, some policy makers have included broader forms of adversity in the conceptualisation of the TIA, such as the Trauma Informed Plymouth Network who discuss ‘Adverse Community Environments’ (Trauma Informed Plymouth Network, n.d.). While such acknowledgements help the policy to address a wider range of experiences, it takes the conceptualisation of the TIA further away from the original idea of addressing ‘trauma’ per se.

Moreover, there are some criticisms regarding some TIA practices. Within the TIA, there is typically some form of screening used to identify trauma and refer for treatment, and that the screening tool is usually the ACE checklist or an adaptation of it (Schulman and Maul, 2019). Notwithstanding the issues of what the ACEs checklist actually measures (as discussed above), one of the authors of the original ACEs study has since argued that it has been misappropriated and misapplied to service delivery and professional practice, cautioning against its use in such a way (Anda et al., 2020). Furthermore, there is evidence that this medicalised model of screening, referring and treating does not sit well with more socially oriented services, with Kerns et al. (2016) finding practitioners feeling uneasy about the use of screening tools to identify trauma. Joy and Beddoe (2019), meanwhile, criticise the ACE tool for not being sensitive to culture, race, poverty and wider issues of power, while Kelly-Irving and Delpierre (2019) argue the ACE tool is not appropriate for individual level assessment.

Linked to these conceptual and operational issues have been criticisms of how a trauma perspective has been implemented into policy and practice (UK Parliament, 2018). Despite existing guidance that has been given on the TIA (e.g., SAMHSA), Donisch et al.’s (2016) research into the opinions and experiences of professionals involved in working in a trauma-informed way found uncertainty about how to actually implement the TIA in practice. Their research found substantial variation in how the TIA was defined and understood among practitioners, and highly idiosyncratic implementations of practices across systems. As they note, there are “varying terms, [a] lack of common lexicon, and differences across systems in knowledge and skills”related to the TIA, and what is lacking is a unified conceptualisation and operationalisation of the approach (Donisch, 2016, p. 131).

The TIA was developed within a specific context to work with people who had most likely experienced trauma. The wider application of this approach to different contexts and more diverse populations, for whom trauma may not be the main issue, inevitably brings complexities and challenges. Conceptual questions are raised about whether ‘trauma’ is the most appropriate lens through which to organise practice and services. Furthermore, there are operational and implementational questions regarding how the TIA is successfully put into practice in a consistent manner that is supported by a robust evidence base. The point is not that the TIA is not a useful way to frame policy and practice, but that it may not be the most effective way to frame all policy and practice for all groups. The question is not just what do we gain by using the TIA, but also what is left out?

In what follows, we discuss how a consideration of shame, along with its impacts and effects, is missing in the TIA. We argue that this omission will be detrimental, leading to the potential ineffectiveness of trauma-informed interventions. As a necessary supplement to any TIA, we argue for the concept and practice of shame-sensitivity.

### Shame

Shame has recently been included in the diagnostic criteria for PTSD in the DSM-V under the umbrella of “persistent negative emotional states” (Taylor, 2015). Hence, shame has recently come to be identified in the trauma literature as part of a constellation of negative emotions (along with fear, horror, anger, guilt) that are common for trauma survivors in post-trauma states. Understanding shame and its role in post-trauma states is, as shall be discussed below, central to the success of the TIA.

Shame is a defining and central feature of human experience and all human relationships, intimately linked to one’s self-perception, social worth, identity, relationships and position within a social group, while also being connected to social control and power through the normative boundaries which determine what is shameful and what is not in a particular society or culture (Dolezal, 2015a, p. 107). Because of its significance and prominence in both personal experience and within social life, shame is considered by many to be the “master emotion” (Scheff, 2004). Shame is commonly characterised as a negative self-conscious emotion; it is an experience that arises when we are concerned about how we are seen and judged by others. We feel shame when we are seen by another or others (whether they are present, imagined or simply a viewpoint that has been internalised) to be flawed in some crucial way, or when some part of our core self is perceived to be inadequate, inappropriate, or immoral.

The term ‘shame’ should be considered an umbrella term that refers to a whole range of experiences, including cognate emotions such as embarrassment, chagrin, mortification and humiliation. As James Gilligan usefully notes, in the same way “that we use the term ‘flower’ as a generic term to refer to a wide variety of different but related plants” then the term ‘shame’ encompasses a wide range of experiences including: “feelings of being slighted, insulted, disrespected, dishonoured, disgraced … demeaned … treated with contempt, ridiculed … mocked, rejected … feelings of inferiority, inadequacy … of being a failure, ‘losing face’, and being treated as if [one is] insignificant, unimportant or worthless” (Gilligan, 2003, p. 1155). What is common to all of these experiences is a sense of being judged negatively by others, and a feeling of being worth less than others.

During a shame experience, we can feel deeply and often irreparably flawed, unworthy and unlovable, and that our social position and our social bonds are under threat. Shame can provoke powerful feelings of despair, inferiority, powerlessness, defectiveness and self-contempt, to name a few. In addition, shame itself is shameful and taboo. As such, shame is an “iterated emotion,” (Dolezal and Lyons, 2017, p. 258); its experience can lead to an intensification or multiplication of itself, leading to a “feeling trap” (Herman, 2011, p. 266) where “one can become ashamed because one is ashamed” (Taylor, 2015). For these reasons shame is usually avoided, shunned or kept secret at all costs, both individually and collectively.

While shame is a negative experience for an individual, it is an inevitable and necessary part of human life. Healthy shame can lead to the expression of positive attributes such as modesty, humility and gratitude, along with respect for oneself and for others. It can also be a powerful motivating force for personal growth and change, and in forging harmonious and meaningful relationships with others (Ng, 2020; Sanderson, 2015). However, healthy shame is very easily distorted and can become ‘unhealthy’, “maladaptive” or “destructive” (Sanderson, 2015, p. 22). As John Bradshaw notes, “shame as a healthy human emotion can be transformed into shame as a state of being… [which] is to believe that one’s being is flawed, that one is defective as a human being. [Shame] becomes toxic and dehumanising” (Bradshaw, 2005, p. xvii). Toxic shame, Sanderson notes, “paradoxically severs connections, destroys social bonds and can lead to antisocial behaviour” (Sanderson, 2015, p. 22). Toxic shame is corrosive and pernicious, and can lead to a pervasive and enduring sense of inferiority, inadequacy, defectiveness, along with a sense of not being worthy of respect, love or connection. It is an experience that can be organise one’s self, life and world, having a deep significance and impact on an individual and their life chances.

A typical shame response involves being overwhelmed with an intense feeling of conspicuousness and a strong sense of being judged by others, along with painful and negative emotions centred around one’s feelings of inadequacy, all triggered by a mishap, mistake or transgression which has been ‘witnessed’ by others (whether they are present, imagined or internalised). This sort of shame response is commonly called “acute shame” (Dolezal, 2015a), insofar as it is a discrete emotional reaction in response to a trigger or event. In contrast, the toxic or pathological shame described above has a very different phenomenological profile, usually occurring in a chronic form. While chronic shame shares many of the painful features of acute shame, such as emotional pain, self-consciousness, a sense of visibility, it is not experienced as a discrete reaction of emotional torment and hyper-self-consciousness. Nor, as the term might imply, is it a state of perpetually feeling shame. Instead, chronic shame is frequently characterised, firstly, by the nagging and persistent *possibility of shame*, and secondly by a persistent sense of inadequacy, defilement, failure and lesser self-worth. Chronic shame can be characterised by what Leon Wurmser terms a “shame attitude” (Pattison, 2000, p. 85), where one’s entire personality and character is structured around shame and shame avoidance.

Chronic shame is an elusive experience for several reasons. First, while ‘chronic shame’ is a term that appears in psychological, psychiatric and psychotherapeutic literatures, there is no clear definition of what constitutes chronic shame and it has been described through a variety of terms including “dispositional shame,” (Leeming and Boyle, 2004) “shame-proneness” (Harris-Perry, 2011), “toxic shame,” (Bradshaw, 2005) and being “shame-based” (Lloyd and Sieff, 2015), among others. There is no clear epidemiological data regarding the prevalence of chronic shame, nor is there any clear diagnostic criteria through which individuals can be ‘diagnosed’ as suffering from chronic shame, or understand their ‘symptoms’ to be mild, moderate, serious or severe (Pattison, 2000, p. 96).

Second, chronic shame is commonly characterised by the nagging and persistent *possibility of shame*, where, for the most part, shame itself is not necessarily realised in experience. Instead, what comes to dominate experience is a pernicious form of anticipated shame, or a persistent and heightened “shame anxiety,”of which an individual may, or may not, be aware (Dolezal, 2021; Pattison, 2000). Shame anxiety appears in experience as a corrosive, undermining and persistent fear or anxiety about being objectified, judged, labelled and rejected by others; it is a persistent “fear of disgrace and being looked at by others with contempt” (Wilson et al., 2006, p. 125). This shame anxiety ultimately becomes connected to negative self-beliefs and self-conceptions; one comes to believe that the “core-self is defective, inadequate and unacceptable to others” (Sanderson, 2015, p. 24). It is important to note that shame anxiety may not be experienced as shame. Instead, it may be dominated by shame avoidance and, as such, characterised by emotions such as fear, anxiety, self-consciousness, stress or powerful impulses to hide, avoid or escape, along with negative feelings about the self, characterised by a sense of inadequacy, defilement or deficiency in relation to others.

While chronic shame has many causes (e.g., societal expectations, stigma and discrimination, psychopathology), it is clear that a significant cause of persistent chronic shame is trauma, where childhood relational trauma and traumatic experiences in later life are strongly correlated with experiences of chronic shame and shame anxiety (DeYoung, 2015; Kalsched and Sieff, 2015; Pattison, 2000). There is also evidence that chronic shame plays a role in PTSD symptom severity (Cunningham, 2020; La Bash and Papa, 2014; Lee et al., 2001). In fact, common defensive scripts or shame-avoidant behaviours seen among those who live with maladaptive chronic shame “bear a strong resemblance,” as Taylor notes, “to the prominent symptoms and behaviours” associated with PTSD (Taylor, 2015). And many experiences related to shame, such as chronic rumination, flashbacks, emotional avoidance, intrusions, hyper-arousal, dissociation and fragmented states of mind are similar to experiences associated with trauma and post-trauma states (Budden, 2009, pp. 1035–1036; Theisen-Womersley, 2021, pp. 210–211).

### Shame and trauma

There is a growing literature that explores the centrality of shame for individuals who have experienced trauma (Budden, 2009; Cunningham, 2020; DeYoung, 2015; Goldblatt, 2013; Herman, 2011; Lee et al., 2001; Øktedalen et al., 2014; Plante et al., 2022; Saraiya and Lopez-Castro, 2016; Sieff, 2015; Taylor, 2015; Theisen-Womersley, 2021; Wilson et al., 2006). Trauma research has seen the recent development of the idea that “shame and trauma are inextricably linked” (Theisen-Womersley, 2021, p. 211), where some argue that “post-traumatic shame” is a key experience that shapes post-trauma states (Theisen-Womersley, 2021), while others have come to theorise and describe PTSD as a “shame disorder” (Herman, 2011; Salter and Hall, 2020), with evidence demonstrating that chronic shame plays a role in PTSD symptom severity (Cunningham, 2020; Lee et al., 2001). Overall, this body of research argues that shame is a world-organising affect for many trauma survivors and that shame is behind much of the maladaptive behaviour associated with trauma, PTSD and other post-trauma states.

The cause of shame in post-trauma states is complex, but there seem to be a multitude of overlapping factors which render shame a predominant, if not the dominant, emotional experience following trauma. Research demonstrates that shame can brought on by: the traumatic experience itself (Budden, 2009; Lloyd and Sieff, 2015); incorrect or inaccurate feelings of blame or responsibility for what happened in the traumatic event (e.g., “it was my fault…”, “this wouldn’t have happened if I had just…”) (Bhuptani and Messman, 2021; Kalsched and Sieff, 2015; Wilson et al., 2006); feelings of defilement and unlovability as a result of neglect or abuse, particularly in childhood (Pattison, 2000); rumination about one’s behaviours, actions and reactions at the time of the trauma (Lee et al., 2001); the sense of being damaged or defiled as a result of having experienced trauma or having a trauma diagnosis, such as PTSD (Herman, 2011); the symptoms of PTSD or a post-trauma state (Lee et al., 2001); the labels attached to one’s identity as a result of trauma and post-trauma outcomes (e.g., “victim”, “survivor”, “addict”, “homeless”) (DeYoung, 2015; Theisen-Womersley, 2021); the coping mechanisms one engages in to cope with trauma (Herman, 2011; Taylor, 2015); fear of judgement by others if they discover one’s trauma (Øktedalen et al., 2014); the social taboos associated with the trauma that one has experienced (e.g., childhood sexual abuse by a family member) (Banaj and Pellicano, 2020); revealing trauma in clinical and psychotherapeutic encounters (DeYoung, 2015; Goldblatt, 2013; Lanksy, 2000); falling short of one’s own ideals and standards (Goldblatt, 2013; Kalsched and Sieff, 2015); and because of the taboo and shameful nature of shame itself (Herman, 2011; Taylor, 2015; Wilson et al., 2006). Hence, in addressing the impact of emotions for trauma survivors, for the treatment of PTSD, and within the TIA, Taylor’s question “have we failed to see the obvious?” with respect to “the influence of shame on posttrauma disorders” seems particularly pertinent (Taylor, 2015).

Understanding shame, and in particular chronic shame, as a keystone sequela of trauma experiences has the potential to elucidate the root cause of a range of maladaptive behaviours associated with trauma. The lack of trust and empathy within intersubjective encounters suggested by some to be characteristic of trauma survivors (Wilde, 2019) are accounted for affectively through understanding shame as central to post-trauma states. However, as noted above, chronic shame is difficult to identify and ‘diagnose’; it is an elusive experience that is often ‘disguised’ or ‘camouflaged’ by other experiences and feelings. The relational psychotherapist Patricia DeYoung notes that what those who suffer from chronic shame, “may not daily or consciously expect to be annihilated by shame. However, the threat is always around somewhere, just out of awareness, kept at bay” (DeYoung, 2015, p. 19). DeYoung describes chronic shame as “silent,” where some of her clients who suffer from chronic shame do not even know that they are anticipating shame (and related strategies to avoid shame) with debilitating frequency. What they live with is not shame, but “what it costs them to keep from falling into shame” (DeYoung, 2015, p. 19). Bradshaw concurs writing that for those living with toxic shame, “everything is organised around preventing exposure” (Bradshaw, 2005, p. 139). As a result, what characterises the experience of chronic shame in post-trauma states is not enduring or repetitive experiences of shame but rather an atmosphere of anticipated shame, or shame anxiety, that leads to compensatory behaviours or experiences.

In this way, in experiences of chronic shame, shame *itself* often becomes invisible and what dominates experience is other behaviour or feelings which are used to help circumvent or avoid shame, or to mask or cope with the pain of shame. As Pattison notes, individuals who experience chronic shame “live their lives trying to avoid occasions and relationships that might provoke painful shame experiences” (Pattison, 2000, p. 83). DeYoung concurs: “the pain [of shame] can be unbearable. To save ourselves, we push shame away as fast as we can, covering for it with more tolerable states of being” (DeYoung, 2015, p. xii). Helen Block Lewis discusses this experience as “bypassed shame” (Lewis, 1971), where the self is not conscious of feeling shame directly, and instead bypasses or ‘displaces’ shame for other emotions, states or experiences (Brown, 1998, p. 146).

As a result, living with chronic shame can lead to a range of compensatory behaviours; these are powerful “defensive scripts” (Kaufman, 1993, p. 113; Pattison, 2000, p. 111), “strategies” (Sanderson, 2015, p. 24) or patterns and habits of interaction, which make it possible for an individual to avoid the social threat, pain and emotional anguish that comes with shame and its chronic anticipation. Lanksy links these to the experience of living with trauma, stating the “posttraumatic state gives rise to shame and to defences that keep shame arousing awareness from consciousness” (Lanksy, 2000, p. 133). Wilson et al. concur, noting that, “the powerful emotions of posttraumatic shame … are associated with a broad range of avoidance behaviours: isolation, detachment, withdrawal, hiding, nonappearance, self-imposed exile, cancellation of appointments, surrender of responsibilities, emotional constriction, psychic numbing, emotional flatness, and non-confrontation with others” (Wilson et al. 2006, p. 138). These avoidance behaviours help an individual protect themselves from shame through avoidance, or “by placing it outside of conscious awareness” (Sanderson, 2015, p. 24). In this way, shame can, as Wilson et al. note, “operate unconsciously in trauma complexes and initiate self-destructive and self-defeating modalities of behaviour” (Wilson et al., 2006, p. 129). Hence, instead of shame, what is seen externally are other reactions, responses and behaviours that “mask the shame” (Ng, 2020, p. 30).

The psychiatrist Donald Nathanson theorises “the compass of shame”, where shame-avoidance behaviours follow four common patterns: withdrawal, avoidance, attack other and attack self (Nathanson, 1992, pp. 305–377). Common defensive behaviours include a variety of different reactions, all of which are damaging both to oneself and to one’s social bonds, such as anger, aggression, hostility, violence, narcissism, depression, perfectionism, apathy, withdrawal, avoidance, excessive deference, among others (Nathanson, 1992; Pattison, 2000). These common defensive reactions to shame are, as Taylor notes, “consistent with many of the symptoms and comorbidities of PTSD” and post-trauma states, including anger, violence, addiction, isolation, feelings of hopelessness and helplessness which can progress to depression and even suicide ideation (Taylor, 2015). What becomes problematic in understanding and treating trauma and the post-trauma states is that these avoidance behaviours for shame are “easily misread” (Theisen-Womersley, 2021, p. 212) and shame often becomes invisiblized and, consequently unacknowledged, in efforts to provide care, treatment and support.

In fact, it has been demonstrated that shame is a “potent treatment barrier” for trauma survivors (Saraiya and Lopez-Castro, 2016), leading to outright avoidance, and to dropping out and attrition once engaged with care and services. As Plante et al. note, shame “generates an urgent need to hide and conceal the defective self from exposure” (Plante et al., 2022). Indeed, there is ample evidence that the ‘necessity’ to avoid shame or shameful exposure can interfere with individuals accessing healthcare (Dolezal, 2015b; Dolezal and Lyons, 2017; Lazare,1987), and also prevent individuals from reporting traumatic incidents such as abuse, sexual assault and violence (Hlavka, 2017; Weiss, 2010). In addition, shame prevents the reporting of shame itself, as individuals “in clinical settings are sometimes reluctant to disclose feelings of shame out of fear from being exposed and rejected” (Øktedalen et al., 2014, p. 600). In these complex and overlapping ways, shame experiences lead to concealment and avoidance, consistent with the “hallmark symptoms” of PTSD and post-trauma states (Saraiya and Lopez-Castro, 2016).

Hence, in the context of seeking help through health, care or social services, individuals who are chronically anxious about shameful exposure may avoid seeking help in the first place, may regularly miss appointments, may avoid disclosing honest details about traumatic events, lifestyle or circumstances, may fail to follow through with treatments, and may conceal diagnoses and coping behaviours from friends, family and professionals (Dolezal and Lyons, 2017). In fact, not only is shame a barrier to accessing services, it is very easily exacerbated and incited in the context of seeking help from professionals; professional practice and public policy are frequently “vectors of shame, humiliation, and inequality” (Salter and Hall, 2020, p. 10). Moreover, shame is a relational emotion that is frequently present in clinical and care encounters (Dolezal, 2015b; Lazare, 1987). Interactions with care professionals can compound feelings of shame, as these interactions often involve unequal power relationships, a fear of being judged, the scrutiny and exposure of one’s potentially ‘shameful’ past, circumstances, lifestyle, coping behaviours, body, illnesses, along with other vulnerabilities. Despite shame’s ubiquity and its obvious impact in encounters with health and care professionals, there is evidence that addressing shame is routinely avoided in clinical and therapeutic encounters, as practitioners themselves are reluctant to acknowledge shame or address experiences which may lead to shame or embarrassment (Lewis, 1971).

It seems clear that being attuned to experiences of shame and chronic shame, along with the common ‘scripts’ and ‘strategies’ deployed to avoid shame and shameful exposure, becomes central to achieving trauma-informed practice, and in fact central to facilitating individuals to seek help and engage with health, care and social services. However, a consideration of shame, along with its impacts and effects, has not been part of the conceptualisation of the TIA, nor an explicit focus in its practice. Indeed, shame is rarely even mentioned in the academic and grey literature about the TIA.

To address this lacuna, we argue for shame-sensitivity to be central to the theory, policy and practice of any TIA. However, the relevance of shame-sensitivity is by no means limited to the TIA. As everyone experiences shame or is vulnerable to shame, shame-sensitivity is of general benefit to all populations and provides a unified framework for good care when working with people more humanely. We do not argue that shame-sensitivity should replace a ‘trauma lens’. Rather we argue that shame-sensitivity, and using a ‘shame lens’, is both necessary for, and has wider application than, the TIA.

### Shame-sensitivity

Shame-sensitivity is a concept and practice for health and human services. There are three central components to the concept. The first is that shame is inevitable. We all have the capacity to experience shame (with a debate about a very small number of individuals (Kosson et al., 2015)), while many vulnerable people live with chronic shame. Interactions with services can, and often do, evoke shame in the people who engage with those services. Second, because shame is a highly unpleasant experience, humans have evolved and developed strategies to avoid shame, and these strategies influence an individual’s thoughts, behaviours and social interactions, usually for the worse. Third, it is incumbent upon services that work with people to acknowledge and respond appropriately to people’s shame in order to mitigate its potential negative effects and impacts. In other words, services need to be shame-sensitive.

While there are a variety of ways to implement shame-sensitivity in practice, and these should be tailored to the specificity of the service provision in question, we outline three key principles for shame-sensitive practice, which we refer to as the 3As: acknowledging shame, avoiding shaming, and addressing shame.

### Acknowledging shame

#### Individual understanding of shame

Practitioners working in human services must have ‘shame competence’. They must have a theoretical and practical understanding of what shame is, how it operates, how it is evoked, how it can be hidden, and understand the behaviours that are used to cope with shame. Not only must individual practitioners be sensitive to the experience of shame in others, but they must also be sensitive to shame within themselves, understanding how shame experiences can affect their own thinking, actions, behaviour and attitudes towards others. Practitioners must also have an understanding of how shame circulates between individuals and within organisations, and also be able to understand when shaming is present in policy and practice.

#### Organisational understanding of shame

Individual shame competence cannot take place without a system of support that accepts the existence, importance, and significance of shame; both for the practitioners themselves and for patients/ clients/service users. This involves the fostering of emotional communication within professional practice, where speaking about and understanding emotions, and their effects, within professional practice becomes commonplace (Gibson, 2014). In particular, the taboo regarding shame, and shameful or stigmatised states and experiences, must be directly addressed. An organisational perspective not only recognises the possibility for the evocation of shame by individuals but also the possibility that organisational policies and procedures can evoke shame in staff and patients/clients/service users.

#### Appreciating the differential experience of shame

significant part of individual acknowledgement of shame is understanding how people come to experience shame, knowing that the boundaries for what is considered shameful can vary for individuals and for different groups. There are variable pressures, standards, contexts, histories and expectations placed on individuals and groups, which can result in shifting signification of what is considered ‘shaming’ or ‘shameful’. By ensuring there is meaningful engagement and collaboration with different communities and groups to understand their particular sensitivities to shame, along with common behavioural responses to avoid the experience of shame, organisations can support individual and collective knowledge and understanding.

#### Recognising shame and shaming

Acknowledging shame moves beyond knowledge of shame theory to also include being able to recognise shame and shaming in experience and practice. Not only is shame frequently hidden and notoriously difficult to admit to, but it is also taboo and shameful. People go to great lengths to hide shame and what they consider to be shameful. Practitioners and organisations must become adept at using a ‘shame lens’ to identify shame through bothphysiological, psychological and social indicators. Practitioners must become aware of common verbal, paralinguistic, and nonverbal cues that may indicate a shame state (Gibson, 2015; Herman, 2011; Retzinger, 1995). These include postural and embodied cues (e.g., covering the face, blushing, downcast eyes, etc.), common terms used instead of shame (e.g., ‘selfconscious’, ‘embarrassed’, ‘foolish’, ‘worthless’, ‘inept’, ‘inferior’, etc.), paralinguistic cues (e.g., stammering, silence, long pauses, etc.). Practitioners must also become adept at recognising bypassed shame, through knowledge and recognition of common avoidance behaviours for shame (cf. ‘the compass of shame’). Practitioners must also become alert to shame dynamics within interpersonal encounters, recognising that shame is a “two-way street” and “contagious” (Theisen-Womersley, 2021, p. 212). This means it can transfer from client, patient or service user to the practitioner, infecting an entire interaction. Practitioners must also have an understanding of how shame circulates within professional organisations and institutions and be able to identify, and also address, implicit and explicit shaming in policy and practice.

### Avoiding shaming

- *Avoiding individual shaming:* Any individual can explicitly seek to shame another person, whether this is a manager to manager, manager to employee, employee to manager, employee to employee, employee to patient/client/service user. With knowledge and understanding of shame and shame dynamics, individuals within a shame-sensitive organisation, practising shame-sensitivity, would actively seek to avoid shaming others. However, they should also be sensitive to the potential for implicit shaming, recognising that any relationship where there are power differences can be inherently shame-inducing (Dolezal, 2015b; Lazare, 1987; Ng, 2020). Individuals engaging with services are expected to expose their vulnerabilities (including their physical bodies, their lifestyle, their illnesses, mental health status, and potentially share intimate details about their past, their families, their feelings etc.), which are then the subject of scrutiny and professional assessment. Practitioners must remain alert to, and continuously assess, how the language they use, their demeanour, questioning style, emotional expression and other interpersonal dynamics may inadvertently produce a shame response (Ford et al., 2021). Furthermore, consideration must be given to interpersonal dynamics, based on gender, race, ethnicity, language-spoken, disability, age, religious identification, along with other factors in particular situations (e.g., a female police officer may be the most ‘shame appropriate’ practitioner to interact with a female victim of sexual assault). Practitioners should also avoid stereotyping, labelling and other stigmatising ways of engaging with individuals. It is imperative to remain responsive to individuals and their unique circumstances and to genuinely acknowledge distress.
- *Avoiding collective shaming*: Many initiatives rely on shame as the affective driver of the change they hope to promote (e.g., shame is frequently used in public health campaigns, for example, to combat obesity or improve hygiene (Brewis and Wutich, 2019)). Such shaming attempts are examples of how whole groups of people can be targets for shame. While there are some initiatives that have an explicit aim to shame groups of people, there are many other initiatives, policies and procedures that have the effect of shaming groups of people, even when this is not intended. Avoiding collective shaming involves being alert to how shaming may become implicit within policy and practice, for instance through the use of stigmatising language, or through creating dynamics of blame and individual responsibility for circumstances or conditions that may be resulting from structural conditions (e.g., poverty, obesity) or that may stem from a post-trauma coping behaviour (e.g., addiction, mental ill health).
- *Evaluating impact of practice for shaming:* Not all proactive attempts to avoid shaming will be successful. To ensure that there is a reflexive feedback system to inform the proactive shaming avoidance attempts, organisations and practitioners must conduct and engage in a process of ongoing evaluation of the impact of their practice, policies, and procedures on the people they come into contact with; both within (employees) and without (patients/clients/ service users) of the organisation (Dolezal et al., 2021). This involves vulnerability, and requires critical reflection on past and future practice. There must be willingness to admit mistakes, openness to critical reflection and flexibility to make responsive changes in policy and practice. Furthermore, organisations must create and systematise nuanced and collaborative understandings of how shaming is produced, and how shame is experienced, as a result of their policies and practices, avoiding attributing blame and shame to individuals where there is a disconnect between policy and operational capacity, especially in cases of chronic underfunding. Collective accountability for shame-sensitive or shame-reducing practice begins with mutually-agreed goals and frames of reference; such as an institutional code of conduct, or a shame-proofing toolkit (Dolezal et al., 2021). Cultures and practices of shaming and blaming must be avoided within organisations (Creed et al., 2014). Cultures of dignity, openness, learning and emotional intelligence should be fostered.

### Addressing shame

#### Addressing individual shame

Being able to address individual experiences of shame requires an understanding of how and why a person experiences their shame and finding ways to work through or around it. This, firstly, means understanding the person in their context and personal history, which will highlight the reasons for the shame experience. Secondly, it necessitates creating a sense of emotional safety (Gibson, 2019), where individuals feel able to talk about their experiences without fear of judgement, criticism, or ridicule, and also with a belief they will be understood and accepted for sharing their feelings. Thirdly, issues related to the experience of shame must be directly discussed in an empathetic and sensitive manner. Language and terminology must be carefully chosen, as the term ‘shame’ can itself be shame-inducing. Alternative phrasing might be more appropriate (e.g., ‘feeling judged’, ‘feeling self-conscious’, ‘embarrassment’, etc.). Unacknowledged and unspoken shame can give the “toxic beliefs that are inherent in shame” some legitimacy (Gibson, 2015, p. 339) and bringing these beliefs out in the open provides the opportunity to unburden the person from shame and reduce the influence it has on interactions. Furthermore, such sensitive discussion of shame requires attentiveness to the person’s needs for support and connection after sensitive disclosures of shame or shame-inducing states, events or circumstances.

#### Supporting shame resilience

While attempts to address shame can occur in any interaction, the effects of shame and disclosing shame can have longer term consequences (Dearing and Tangney, 2011). The experience of shame can leave individuals to “feel isolated … and shy away from reaching out to people who may be able to offer help for fear of rejection and further shame” (Gibson, 2015, pp. 339–340). Shamesensitive practice, organisations, and systems, therefore, need to embed shame resilience into the ways they address shame. At the heart of shame resilience is the development and deepening of social bonds (Brown, 2006). It is imperative that practitioners engage in practice that creates and promotes sustainable relationships with and within any organisation (Gibson, 2015). Organisations and services need to ensure continuity with individual practitioners so meaningful relationships grounded in familiarity, trust and empathy can be developed. Practitioners and services need to be proactive in reaching out to individuals, especially when they disengage. Individuals should not be made to feel cut off, disconnected or discarded from services. Structural factors such as the availability of appointment times, accessibility of clinical spaces, ease through which one can contact the service, length of waiting lists, duration of service, continuity between services, must be continually assessed to ensure that individuals feel supported and a sense of connection is maintained. Furthermore, friend and family networks must be supported so that individuals have sustainable networks of support. In addition, practitioners must be supported by their organisations and institutions to have the time, support and resources to engage in genuinely relational practice, fostering connection, empathy and trust with the individuals they are working with and supporting.

#### Actively fostering the conditions for shame-sensitive practice

Organisations must actively work to create the conditions, policy and practices that promote shame-sensitivity, where relationships based on dignity, respect, empathy and trust are the first priority within workplaces and when delivering services. Practitioners must be supported within organisations to have the personal, professional and operational capacity to work in a shame-sensitive manner.

#### Combating the systemic causes of shame

The systemic forces which shape and define what is considered shameful or stigmatised are not immutable. In addition, many causes of trauma (e.g., social deprivation, domestic abuse) have their roots in societal and structural conditions which can be changed and improved. Practitioners, along with leaders and managers within organisations, must be given the resources and encouraged to be engaged in making meaningful changes. This will happen through creating cultures of engaged practice and political activity, where individuals are encouraged to write to local councillors or Members of Parliament, carry out research, engage with academic partners, become involved in local and national political campaigns, engage with media outlets, etc., with the overall aim of advocating and agitating for more humane and shame-sensitive changes in law, policy and practice (Gibson, 2019, p. 199).

## Conclusions

Having the capacity, on the levels of policy, organisations and individual practitioners, to address shame directly is imperative considering the how impactful shame can be for those who have experienced trauma and post-trauma states. Being attentive to shame, and acknowledging its significance for individuals, in health and social care contexts, can improve both engagement and outcomes. Using a ‘shame lens’ alongside a ‘trauma lens’ is necessary for TIAs to achieve the goal of redesigning services to be more sensitive and supportive, with the ultimate aim of avoiding retraumatisation and any additional harm. As a result, TIAs must begin to integrate shame-sensitive practice. There are obvious overlaps and synergies with the main principles which guide TIAs, however focusing through a ‘shame lens’ will reveal significant affective dynamics that are otherwise occluded, overlooked or ignored.

Shame-sensitivity and using the ‘shame lens’ within organisations will enable more humane services which address and acknowledge a significant affective dimension of seeking help, namely shame and self-consciousness. Following the evidence that shame is a significant force within encounters with professionals within health, care and social services, introducing a ‘shame lens’ to the way these services are conceptualised and conducted, has the potential to transform interactions between professionals and patients/clients/service users, as well as among colleagues within services and organisations. The emotional intelligence that shame-competence affords will give practitioners greater awareness of social dynamics which will help manage interactions and relationships within encounters with more empathy, humanity and sensitivity. Having more awareness of emotions and emotional dynamics within workplaces has been linked to a range of positive outcomes, such as ability to handle stress, improved job performance, job satisfaction and leadership skills (Magny and Todak, 2021, p. 958). Understanding shame, in particular, can uncover and unlock a range of usually occluded dynamics between individuals and within institutions that have negative or damaging effects (Creed et al., 2014).

While shame-sensitive practice is essential for the TIA, it should be acknowledged that shame is a universal experience, and that shame-sensitive practice should be integrated into all service delivery, and not just seen as an accompaniment to trauma-informed care. All individuals experience shame, and this can be easily exacerbated in contexts where there are unequal power relations, such as in encounters with doctors, social workers, police and other health and care professionals. In addition, shame-sensitive practice is not intended to be a solution for the social ills that lead individuals to need to engage with services. The integration of this approach must be within broader societal efforts to reduce conditions that produce chronic shame, stigma and trauma, such as poverty, destitution, deprivation, long-term unemployment, violence, sexual assault, domestic abuse, displacement, etc. These principles for practice will be most effective in environments that have long-term viability and also are also well-resourced, where there is also widespread public confidence in services and organisations.

Offering an outline of the concept and the practice of shame-sensitivity, this article has highlighted what is needed for human services to effectively face shame and shaming and mitigate their negative impacts and effects. We argue that principles of shame-sensitivity, and the practice that goes along with it, are the starting point for any interactions, organisational changes, and policy developments. The corollary of this is that these principles and practices should precede a TIA, that they will address many of the issues that people face following trauma, but where additional care and support is needed these principles should be integrated into the TIA.
